# Epigallocatechin Gallate-Modified Silver Nanoparticles Show Antiviral Activity against Herpes Simplex Type 1 and 2

**DOI:** 10.3390/v15102024

**Published:** 2023-09-29

**Authors:** Malgorzata Krzyzowska, Martyna Janicka, Marcin Chodkowski, Magdalena Patrycy, Oliwia Obuch-Woszczatyńska, Emilia Tomaszewska, Katarzyna Ranoszek-Soliwoda, Grzegorz Celichowski, Jaroslaw Grobelny

**Affiliations:** 1 Military Institute of Hygiene and Epidemiology, Kozielska 4, 01-163 Warsaw, Poland; martyna.janicka@wihe.pl (M.J.); marcin.chodkowski@wihe.pl (M.C.); magdalena.patrycy@wihe.pl (M.P.); o.woszczatynska@gmail.com (O.O.-W.); 2 Division of Microbiology, Department of Preclinical Sciences, Institute of Veterinary Medicine, Warsaw University of Life Sciences, 02-786 Warsaw, Poland; 3 Department of Materials Technology and Chemistry, Faculty of Chemistry, University of Lodz, Pomorska 163 St., 90-236 Lodz, Poland; katarzyna.soliwoda@chemia.uni.lodz.pl (E.T.); emilia.tomaszewska@chemia.uni.lodz.pl (K.R.-S.); grzegorz.celichowski@chemia.uni.lodz.pl (G.C.); jaroslaw.grobelny@chemia.uni.lodz.pl (J.G.)

**Keywords:** HSV-1, HSV-2, epigallocatechin gallate

## Abstract

(1) Background: Epigallocatechin gallate (EGCG) has been recognized as a flavonoid showing antiviral activity against various types of DNA and RNA viruses. In this work, we tested if EGCG-modified silver nanoparticles (EGCG-AgNPs) can become novel microbicides with additional adjuvant properties to treat herpes infections. (2) Methods: The anti-HSV and cytotoxic activities of EGCG-AgNPs were tested in human HaCaT and VK-2-E6/E7 keratinocytes. HSV-1/2 titers and immune responses after treatment with EGCG-AgNPs were tested in murine models of intranasal HSV-1 infection and genital HSV-2 infection. (3) Results: EGCG-AgNPs inhibited attachment and entry of HSV-1 and HSV-2 in human HaCaT and VK-2-E6/E7 keratinocytes much better than EGCG at the same concentration. Infected mice treated intranasally (HSV-1) or intravaginally (HSV-2) with EGCG-AgNPs showed lower virus titers in comparison to treatment with EGCG alone. After EGCG-AgNPs treatment, mucosal tissues showed a significant infiltration in dendritic cells and monocytes in comparison to NaCl-treated group, followed by significantly better infiltration of CD8+ T cells, NK cells and increased expression of IFN-α, IFN-γ, CXCL9 and CXCL10. (4) Conclusions: Our findings show that EGCG-AgNPs can become an effective novel antiviral microbicide with adjuvant properties to be applied upon the mucosal tissues.

## 1. Introduction

Epigallocatechin gallate (EGCG) is a flavonoid belonging to the chemical class of flavan-3-ols (catechins) esterified with gallic acid [1,2]. Flavonoids can be found in beverages, vegetables, and fruits, such as tea, coffee, wine, berries etc., and have demonstrated favorable effects against various diseases, including cancer, obesity, diabetes, and neurodegenerative disorders [3,4,5]. EGCG accounts for 50% of THE catechins present in green tea (*Camellia sinensis*) [1,2]. It has been found to be the most potent and universal virus inhibitor among catechins, showing antiviral activity against various types of enveloped DNA [6,7,8,9], (−)-RNA [7,8,9,10], and (+)-RNA viruses [7,8,11]. EGCG inhibits THE early stages of infection, such as attachment and membrane penetration by virions, by interfering with either viral membrane proteins or cellular protein, or both of them. For example, EGCG interacts with the herpes simplex type 1 (HSV-1) glycoprotein gB and gD, which bind to heparan sulfate residues on cellular glycans, thus blocking viral entry [6,7]. EGCG was shown to inhibit hemagglutination induced by the hemagglutinin (HA) envelope glycoprotein of influenza A virus (IAV), demonstrating its ability to compete with IAV binding to sialic acid [7,8]. EGCG inhibits attachment of hepatitis C virus (HCV) [7,9] vaccinia virus (VACV) [7], adenovirus type 5 (AdV-5) [7], reovirus [7], vesicular stomatitis virus (VSV) [7], Zika virus (ZIKV) [10], dengue virus (DENV) [10], West Nile virus (WNV) [10], and human immunodeficiency virus (HIV-1) [11], to the target cells.

EGCG can also exert its antiviral properties by interfering with other steps of the viral replication cycle. For example, EGCG combined with a NS3/4A protease inhibitor or cyclosporine A, showed strong additive inhibitory effect upon HCV replication [9]. In human keratinocytes transfected with type 2 HPV (HPV-2), EGCG pretreatment up-regulated expressions of interferon-stimulated genes (ISGs) and type I IFN signaling antiviral pathway components, which were significantly downregulated by HPV transfection [12]. 

Herpes simplex virus (HSV) causes a recurrent, contagious infection that affects approximately 60% to 95% of adults worldwide. HSV-1 is associated mainly with orofacial infections, while HSV-2 causes mostly anogenital infections. After the initial or primary infection, HSV-1 and -2 persist in the body by becoming latent in the cell bodies of nerves [13]. Antiviral drugs targeting viral replication, such as acyclovir or valacyclovir, can partially control the signs and symptoms of genital herpes when used to treat the first clinical and recurrent episodes. However, these drugs neither eradicate the latent virus nor affect the risk, frequency, or severity of recurrent infections [14]. 

Both silver nanoparticles (AgNPs) and gold nanoparticles (AuNPs) have recently gained interest by exerting an antiviral activity against several viruses [15,16]. There are two ways that the Ag/AuNPs can inhibit the pathogenic virus: (1) they bind to the outer coat of the virions thus blocking virus attachment and cell entry and (2) NPs bind to the DNA or the RNA of the virus intracellularly, inhibiting the replication or propagation of the virus [15,16]. 

We have previously described anti-HSV-2 activity of AgNPs modified by tannic acid (TA-AgNPs) [17,18] or lactoferrin and lactoferrin-modified AuNPs [19] by using both in vitro and in vivo models. The mechanisms of antiviral action of both LF-Ag/AuNPs and TA-AgNPs included blocking of virus attachment, entry, cell-to-cell spread and induction of anti-viral cytokine and chemokine production [17,18,19]. Moreover, the anti-viral effect was dependent on the size of nanoparticles, with smaller nanoparticles showing stronger activation of inflammatory reaction [17,19]. In our study, 30 nm TA-AgNPs improved the anti-HSV-2 immune response by boosting a virus-specific cellular and humoral response with activation of B cells [17,18,19]. 

In this study, we tested whether modification of 30 nm AgNPs with EGCG can lead to development of a novel nanomicrobicide showing both good antiviral and toxicity profiles using in vitro and in vivo models of HSV-1 and HSV-2 infection. 

## 2. Materials and Methods

### 2.1. Synthesis of Modified Silver Nanoparticles

#### 2.1.1. Chemicals

Silver nitrate (AgNO_3_, purity 99.999%, Sigma-Aldrich, St. Louis, MO, USA), tannic acid (C_76_H_52_O_46_, Sigma-Aldrich), sodium citrate (C_6_H_5_Na_3_O_7_·2H_2_O) purity 99.0%, (Sigma-Aldrich), sodium borohydride (NaBH_4_, purity ≥ 96%, Sigma-Aldrich), epigallocatechin gallate (Extrasynthese, Genay Cedex, France) were used as received. For all preparation of aqueous colloids, deionized water obtained from the Deionizer Millipore Simplicity UV system (specific resistivity of water was 18.2 MΩ∙cm) was used.

#### 2.1.2. Silver Nanoparticles

Silver nanoparticles were synthesized according to the seed growth-mediated method. The synthesis procedure included two steps: (i) silver seeds synthesis and (ii) preparation of 30 nm AgNPs using previously synthesized AgNPs seeds. The first step of the synthesis was as follow: sodium citrate (0.228 g) and deionized water (95 g) were mixed and heated at 70 °C for 15 min., silver nitrate (1.7 mL, 1%) was added, and then after 2 s sodium borohydride (2 mL, 0.1%) was added with the syringe pump (syringe capacity 2 mL, diameter 8.00 mm, flow rate 55 mL∙h^−1^). Next, the mixture was additionally heated at 70 °C for 60 min, and then cooled down to room temperature. The synthesis procedure was carried out without reflux, hence after the synthesis, an amount of deionized water was added by weight so that the colloid mass was finally equal to 100 g. The final metal concentration in the colloid was 100 ppm. The second step of 30 nm AgNPs synthesis was as follows: sodium citrate (3 g, 1%) and deionized water (75 mL) were heated for 15 min under reflux. Next, an aqueous solution of silver nitrate was added to the reaction mixture with the syringe pump (1% aqueous solution, 2.6 mL, syringe 10 mL, diameter 10 mm, flow 20 mL∙h^−1^), and silver seeds were added (10 g). The whole mixture was heated for 1 h under reflux. After this time period, the second portion of sodium citrate was added (3 mL, 1%) along with the second portion of silver nitrate with a syringe pump (1% aqueous solution, 2.6 mL, syringe 10 mL, diameter 10 mm, flow 20 mL∙h^−1^), and the mixture was heated for another 60 min and then cooled down to room temperature. The final AgNPs concentration was 355 ppm, and for subsequent experiments this colloid was diluted with deionized water to 100 ppm.

#### 2.1.3. Epigallocatechin Gallate-Modified AgNPs (EGCG-AgNPs)

30 nm AgNPs functionalized with EGCG were prepared by modification of citrate AgNPs from 2.1.2. with epigallocatechin gallate. Briefly, 1 g of AgNPs colloid was incubated with 0.015 g of aqueous EGCG solution (0.611%), then vigorously stirred at room temperature for 60 min. The final concentration of EGCG in colloid was 200 μmol/L (92 ppm).

#### 2.1.4. Tannic Acid-Modified AgNPs (TA-AgNPs)

TA-AgNPs were synthesized as follows: silver nitrate aqueous solution (95.2 g, 0.017%) was heated to boiling point under reflux and next a mixture of an aqueous solution of sodium citrate (4.2 g, 4%) and tannic acid (0.6 g, 5%) was added. The solution was vigorously stirred under reflux for an additional 15 min and cooled down to room temperature. The concentration of nanoparticles in all colloids was 100 ppm and tannic acid had 0.0315 wt.% (315 ppm).

#### 2.1.5. NPs Characterization

The characterization of AgNPs, EGCG-AgNPs, TA-AgNPs was performed using Dynamic Light Scattering (DLS, Litesizer 500, Particle Analyzer, Anton Paar, Graz, Austria), High-Resolution Scanning Electron Microscopy equipped with transmission detector STEM II (HR-STEM, NovaNanoSEM 450, FEI, Hillsboro, OR, USA) and UV-vis spectroscopy (HL-2000, Ocean Optics, Ostfildern, Germany). Samples for HR-STEM were prepared by drop-casting an aqueous dispersion of the nanoparticles onto carbon-coated copper grids and measurements of at least 500 nanoparticles.

### 2.2. Cell Lines

Human VK2-E6/E7 vaginal epithelial cells (ATCC^®^ CRL-2616) and Vero cells (ATCC^®^ CCL-81) were obtained from ATCC (Rockville, MD, USA). VK-E6/E7 cells were maintained in EpiLife™ medium supplemented with EpiLife™ Defined Growth Supplement (EDGS) (Thermo Fisher Scientific, Waltham, MA, USA) and 10 U/mL penicillin and 100 μg/mL streptomycin (GIBCO by Thermo Fisher Scientific). Human HaCaT keratinocytes were purchased from CLS Cell Lines Service GmbH (Eppelheim, Germany). HaCaT and Vero cells were grown in Dulbecco’s modified MEM medium (DMEM) supplemented with 10% fetal calf serum, 10 U/mL penicillin, and 100 μg/mL streptomycin (GIBCO). All cell lines were maintained at standard conditions (37 °C, 5% CO_2_) and handled according to ATCC and CLS recommendations. 

### 2.3. Cell Viability Assay

MTT assay (-(4,5-Dimethyl-2-thiazolyl)-2,5-diphenyl-2H-tetrazolium bromide, Sigma-Aldrich) was used to measure cell viability in the presence of NPs. For all tests, overnight cultures of Vero, HaCaT and VK-2-E6/E7 cells in a 96-well plate with approx. 100% confluence (10^3^ cells/well) were prepared. The cells were incubated at 37 °C with EGCG-modified and unmodified AgNPs in a range of 1–75 μg/mL concentrations in a final volume of 100 µL for 24 h. The control cells were left untreated. Twenty µL of MTT at the concentration of 5 mg/mL in PBS was added to each well, and the cells were incubated for 2 h at 37 °C. Next, 100 µL of the solubilization solution (10% Triton X-100, 0.1 M HCl in isopropanol) was added to each well, and after 30 min of incubation in room temperature, the optical density (OD) was measured at 570 nm using Omega Microplate Spectro-photometer (BioTek Instruments, Inc., Winooski, VT, USA). The cellular viability was defined as the percentage of viable cells relative to the untreated control. All experiments were performed in triplicate.

### 2.4. Virus and Anti-Viral Tests

HSV-1 (strain McKrae) and HSV-2 (strain 333) were grown and titrated in Vero cells and kept at −80 °C until use. 

#### 2.4.1. Plaque Reduction Assay

Vero cells were cultured in DMEM complete medium in 24-well plates until reaching 100% confluence (approx. 10^5^ cells/well). To assess antiviral activity of EGCG-AgNPs, HSV-1 or -2 (100 PFU/mL) were incubated for 60 min with 7.5 μg/mL of EGCG-AgNPs, tannic acid-modified 30 nm AgNPs or EGCG solution (100 μM). The mixture was added to Vero cells for 60 min at 37 °C, after which cultures were washed with cold PBS, and 2% methylcellulose in complete culture medium was added to block spreading of the virus through the cell culture medium. Forty-eight hours later, plates were washed, stained with 1% crystal violet and counted for the number of plaques. Data were expressed as % of infection inhibition, (in %) using the equation; 100 × [1 − (number of plaques with treatment)/(number of plaques without treatment)].

#### 2.4.2. Inhibition of Virus Attachment or Penetration

The antiviral activity of EGCG-modified AgNPs during the stage of virion attachment or penetration were accessed in VK2-E6/E7 and HaCaT. To analyze the influence upon viral attachment, cells were pre-chilled at 4 °C for 15–20 min, then EGCG or TA- modified AgNPs or EGCG previously quickly mixed with 1000 PFU/mL of HSV-1 or HSV-2 were applied to the cell cultures for 1 h at 4 °C. After this time, virus and NPs or EGCG were removed by washing with ice-cold PBS, and further moved to 37 °C. At 24 h post infection, virus titers were quantified in total isolated DNA by qPCR (see below), as described previously [17,18,19]. For penetration assay, cells were pre-chilled at 4 °C for 15–20 min, then infected for 1 h at 4 °C with 1000 PFU/mL of HSV-1 or HSV-2. After this time, cells were washed with ice-cold PBS before adding modified AgNPs or EGCG for 2 h at 37 °C. The cells were afterwards washed twice with cold PBS. After 18 h p.i., total DNA was extracted and used to determine HSV-1/HSV-2 titers by qPCR, as described below.

### 2.5. Primary Keratinocytes and Treatment

Skins from newborn C57BL/6 mice were collected into dispase/PBS solution (1 mg/mL) and incubated overnight at 4 °C. Next day, epidermis was scraped off from dermis using forceps and placed in 1.25% trypsin-EDTA solution and incubated at 37 °C for 10 min. Keratinocytes were collected by centrifuging and further cultured in in Keratinocyte-Serum Free Medium (SFM) with 0.1 ng/mL human recombinant EGF, 0.05 mg/mL bovine pituitary extract, 0.4 mM calcium chloride with 10 U/mL penicillin and 100 μg/mL streptomycin (GIBCO by Thermo Fisher Scientific). After 5 days of culture in 24-well plates, the keratinocytes were used for in vitro experiments. Primary cultures were infected with HSV-1 or HSV-2 at MOI (multiplicity of infection) of 1 for 4 h. Next, unbound virus was washed with PBS and the cells were treated with EGCG-AgNPs or EGCG at 7.5 μg/mL. Treated or untreated uninfected cultures were used as controls.

### 2.6. Ethical Statement

The protocol was approved by the Local Committee on the Ethics of Animal Experiments in Warsaw, Poland (permit Number: WAW2/69/2021), and animal experimentation guidelines were strictly followed.

### 2.7. In Vivo Infection Model

Male or female, 6- to 8-week-old C57BL/6 mice were used for all experiments. Mice were kept under standard environmental conditions. For HSV-1 infection, mice were anesthetized with isoflurane (Baxter, Tramco, Wolskie, Poland), and a total dose of 1 × 10^6^ PFU of HSV-1 in 10 μL was given intranasally. At 24 and 48 h post infection, mice received intranasally 20 μL of 0.9% NaCl solution containing 20 μg/mL of EGCG-AgNPs or 0.9% NaCl solution. 

For genital HSV-2 Infection, female mice were injected s.c. (subcutaneous) with 2.0 mg/mouse of medroxyprogesterone (Depo-Provera; Pfizer, Puurs, Belgium) in 200 µL of PBS. Five days later the mice were anesthetized and inoculated intravaginally with 20 μL of PBS containing 10^3^ PFU of HSV-2 strain 333. Vaginal washings were performed at 24 and 48 h after infection with 100 μL of 20 μg/mL EGCG-AgNPs/0.9% NaCl or 0.9% NaCl solution. The washings were performed with 200 μL pipet tips, by introduction of 100 μL of a liquid, followed by evacuation of the liquid with the same tip, repeated twice (total volume of a vaginal washing—approx. 200 μL).

Mice were monitored daily and those showing neurological symptoms or significant weight loss were immediately removed from the experiment. At day 3 or 7 post infection, mice were sacrificed by cervical dislocation and brains, trigeminal ganglia, and nasal cavities (HSV-1 model) or the spinal cords and vaginas (HSV-2 model) were collected for further tests.

### 2.8. Quantitative PCR 

RNA/DNA Extracol kit (Eurx, Gdansk, Poland) was used to isolate DNA and RNA from vaginal tissues, spinal cords, brains and trigeminal ganglia, and a Universal RNA/DNA extraction kit (Eurx) served to isolate DNA from infected cell cultures and vaginal lavages. HSV-1 and HSV-2 were detected by qPCR with primers and probe for the viral envelope glycoprotein (gB), as described by Namvar et al. [20] in QuantStudio™ 5 Real-Time PCR System (Thermofisher Scientific) with GoTaq^®^ Probe qPCR Master Mix (Promega, Madison, WI, USA). Standard curve analysis was based on Ct values and serial of 10-fold dilutions of the plasmid standard containing the gB gene. A standard curve was included in each PCR run. Data are expressed as the HSV-2 copy number per ng of the total isolated DNA. 

RNA isolated from tissues was converted to cDNA using GoScript™ Reverse Transcriptase (Promega). qPCR reactions for cytokines and chemokines were carried out using GoTaq^®^ Probe qPCR Master Mix (Promega) and TaqMan^®^ probes (Thermofisher) for the detection of IL-1β (Mm00434228_m1), IFN-γ (Mm01168134_m1), IFN-α4 (Mm00833969_s1), TNF-α (Mm00443258_m1), CXCL9 (Mm00434946_m1), CXCL10 (Mm00445235_m1), IL-10 (Mm01288386_m1), IL-6 (Mm00446190_m1) and GADPH (Mm99999915_g1), according to the manufacturer’s instructions using QuantStudio™ 5 Real-Time PCR System (Thermofisher Scientific). Results were analysed with the 2^−ΔΔCt^ cycle threshold (2^−ΔΔCt^) method.

### 2.9. Flow Cytometry Phenotypic Analysis

Cell suspensions from tissues were prepared as follows: brains and trigeminal ganglia were pressed through a 70 µm cell strainer and washed in PBS/1%FBS. Vaginal tissues were cut into small pieces and treated with Liberase TL (Roche, Indianapolis, IN, USA) in MEM medium at 37 °C for 40 min. The nasal cavities were first homogenized with a hand homogenizer. Tissues were next pressed through a 70 µm cell strainer and washed in PBS/1%FBS, as above. To block Fc receptors, cell suspensions were pretreated with rat anti-CD16/32 antibody (2.4G2) (BD Biosciences, Franklin Lakes, NJ, USA) for 10 min on ice. After washing in PBS/1%FBS, the following antibodies were used: anti-CD3-FITC (145-2C11, Thermo Fisher Scientific), anti-CD4-PE (clone RM4-5, BD Biosciences), anti-CD8-PerCP (clone 53-6.7., BD Biosciences), anti-NK1.1-APC (clone PK136, BD Biosciences), anti-CD11b-FITC or PE (clone M1/70) (BD Biosciences), anti-CD86-PE (clone GL1, BD Biosciences), anti-CD206-APC (clone MMR, ThermoFisher Scientific), anti-CD11c-APC (HL3, eBioscience, Cambridge, MA, USA), rat anti-F4/80-FITC (BM8, eBioscience) and rat anti-I-A-PE (M5/114.15.2, eBioscience) antibodies, and rat anti-Gr-1-PE (RB6-8C5, BD Biosciences). Following the immunolabeling for the extracellular markers, cells were fixed with Perm/Wash buffer (BD Bioscience) and were incubated with anti-IFN-γ APC (eBioscience, clone-XMG1.2). The amount of the antibodies used for staining was determined after optimalisation of staining for each type of tested tissue (0.5 or 1 μL of Ab per 10^6^ cells).The stained cell suspensions were analyzed in Becton Coulter CytoflexTM for percentage of positively stained cells and analyzed using FlowJo software (Tree Star, Ashland, OR, USA).

### 2.10. Chemokine and Cytokine Analysis in Vaginal Lavages

Vaginal lavages obtained at 2nd washing were collected and frozen. Chemokine and cytokine analysis was performed with a mouse magnetic Luminex assay from R&D Systems for 23 cytokines/chemokines using Bio-Plex 200 system (Bio-Rad, Hercules, CA, USA). The levels of CXCL9, CXCL10 and IFN-alpha were measured using Mouse CXCL9/MIG Quantikine ELISA Kit (R&D, Minneapolis, MN, USA), Mouse CXCL10/IP-10/CRG-2 DuoSet ELISA kit (R&D) and Mouse IFN-α ELISA Kit (R&D) according to manufacturer’s instructions.

### 2.11. Statistics

Statistical analysis was performed with GraphPad Prism version 7 (GraphPad software). Data were analyzed using an unpaired Student’s *t*-test (normal distribution) or the Mann–Whitney U test and the results are reported as mean ± standard error of the mean (SEM) unless indicated otherwise. The *p* ≤ 0.05 was considered statistically significant.

## 3. Results

### 3.1. Characterization of Nanoparticles

**Figure 1:**
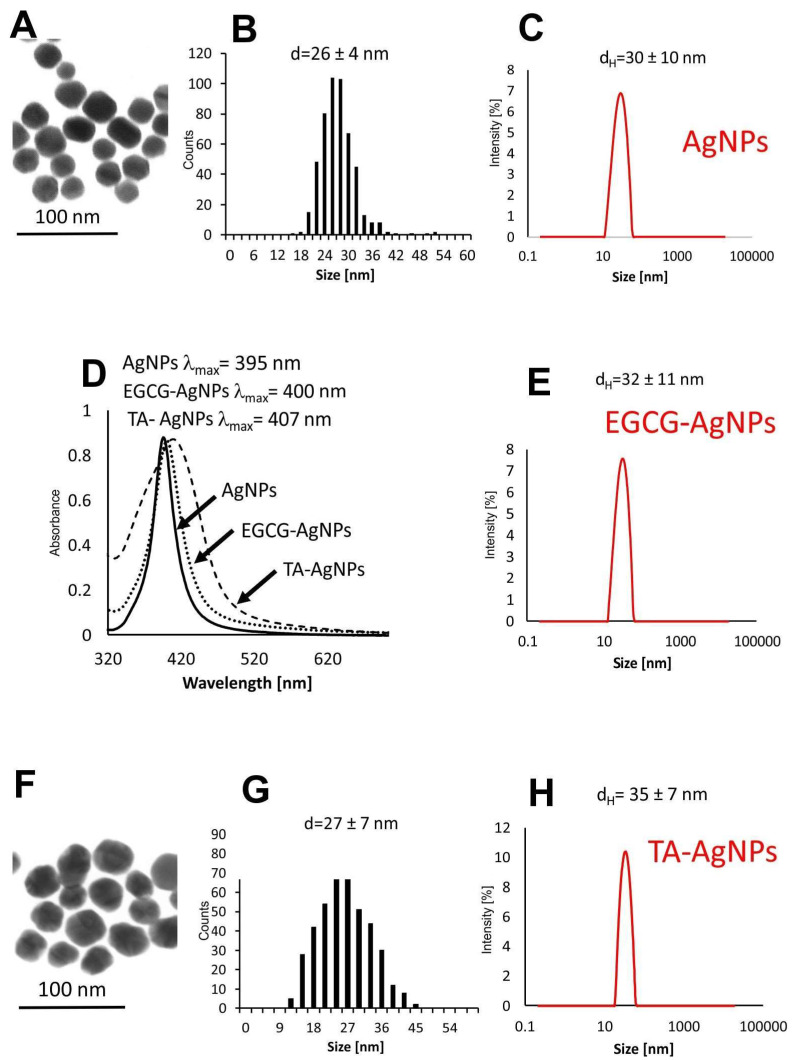
Physicochemical characterization of AgNPs (**A**–**D**), EGCG-AgNPs (**D**,**E**) and TA-AgNPs (**D**,**F**–**H**): HR-STEM (**A**,**F**); size distribution histograms (**B**,**G**); UV-vis spectra (**D**) and DLS size distribution histograms (**C**,**E**,**H**).

Silver nanoparticles (AgNPs, EGCG-AgNPs and TA-AgNPs) were precisely characterized to determine the size, shape, size distribution and colloidal stability of nanoparticles as well as to determine and confirm the stability of nanoconjugates after EGCG functionalization (Figure 1). UV–Vis spectroscopy was used for confirmation of NPs synthesis by observation of the characteristic localized surface plasmon resonance (LSPR) peak in UV–Vis spectra. For all colloids, the required peak was observed in the region characteristic for specific nanoparticles (Figure 1D): for AgNPs λmax = 395 nm, for EGCG AgNPs λmax = 400, for TA-AgNPs λmax = 407 nm. HR-STEM measurement revealed the spherical shape of all nanoparticles with the mean size of metallic core equal: dSTEM AgNPs = 26 ± 4 nm (Figure 1A,B) and dH TA-AgNPs = 27 ± 7 nm (Figure 1F,G). The size of metallic core of EGCG-AgNPs was equal AgNPs (26 ± 4 nm) and this colloid was used for the preparation of EGCG-AgNPs conjugates. The hydrodynamic particle size was equal: d_H_ AgNPs = 30 ± 10 nm (Figure 1C); d_H_ EGCG-AgNPs = 32 ± 11 nm (Figure 1E); d_H_ TA-AgNPs = 35 ± 10 nm (Figure 1H), and Zeta potential equal: ζ AgNPs = −76.0 mV, ζ EGCG-AgNPs = −67.0 mV and ζ TA-AgNPs = −58.0 mV (Table 1).

### 3.2. Anti-Viral Effects of EGCG-Modified AgNPs In Vitro

To ensure that tested EGCG-AgNPs concentrations were non-toxic, cytotoxicity of nanoparticles in Vero, VK-2 and HaCaT cell lines and in primary murine keratinocytes was assessed using the MTT assay (Table 2). The results of the MTT assay demonstrated that EGCG-AgNPs showed better toxicity profiles in comparison to unmodified AgNPs and were within the same range as the results for EGCG (Table 2). The 50% cytotoxic concentrations (CC_50_) and MNTC (maximum non-toxic concentrations) for all three cell lines were also similar (Table 2). Therefore, for in vitro infectious assays, we chose a concentration of 7.5 µg/mL for EGCG-modified or unmodified 30 nm AgNPs. 

**Figure 2:**
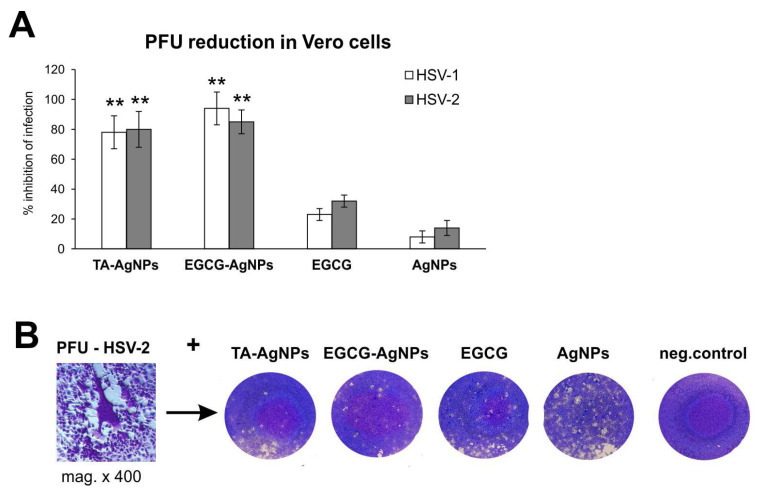
Epigallocatechin gallate-modified AgNPs inhibit HSV-1 and HSV-2 infection in vitro. (**A**) HSV titers (PFU/mL) in Vero cells infected with HSV-1 or 2 pre-incubated for 1 h with TA-AgNPs, EGCG-AgNPs, EGCG and AgNPs. (**B**) Representative micro (**left**) and macro (**right**) photos of crystal violet-stained HSV-2-infected Vero cultures with visible cytopathic effect (PFU) wells corresponding to graphs. The data are expressed as means from three independent experiments ± SEM. ** represents significant differences with *p* ≤ 0.001 in comparison to infected control.

The antiviral properties of EGCG-modified AgNPs were first tested by the standard plaque forming assay (PFU/mL) in Vero cell culture. Incubation of HSV-1 or HSV-2 with EGCG-modified AgNPs for 60 min prior to infection showed significant inhibition of HSV-1 and HSV-2 infection by both TA-AgNPs and EGCG-AgNPs (*p* ≤ 0.001) (Figure 2), and much less efficient inhibition by EGCG solution with concentration corresponding to the amount used to modify NPs (Figure 2). Unmodified AgNPs (Figure 2) showed no significant inhibition of HSV-1 or HSV-2 infection (Figure 2). 

**Figure 3:**
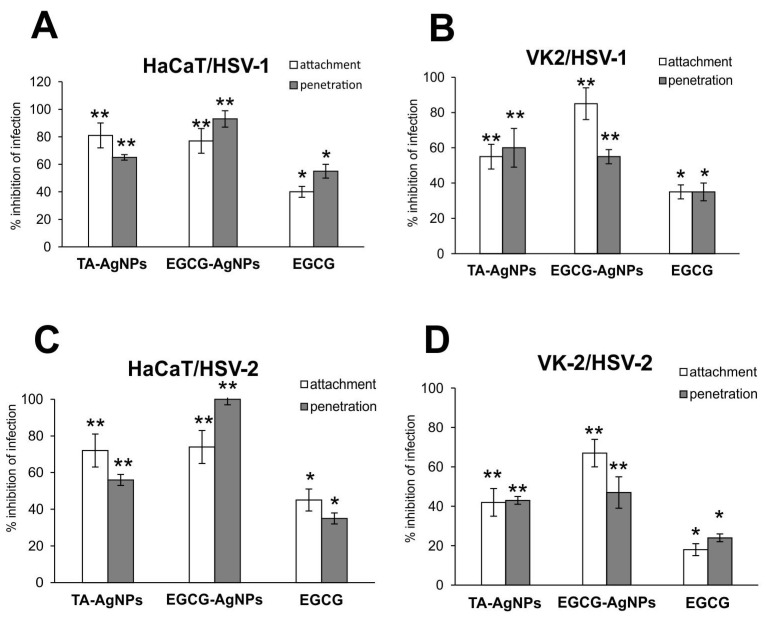
Epigallocatechin gallate-modified AgNPs inhibit HSV-1 and HSV-2 penetration and attachment better then EGCG and TA-AgNPs. Inhibition of virus attachment and penetration in HaCaT (**A**,**C**) and VK-2-E6/E7 (**B**,**D**) cell cultures with the use of TA-AgNPs, EGCG-AgNPs and EGCG. At 24 h post infection (p.i.), the infected cells were subjected to HSV-1/2 copies titration by qPCR. The data are expressed as means from three independent experiments ± SEM. * represents significant differences with *p* ≤ 0.05, ** *p* ≤ 0.01, in comparison to untreated infected control.

Since EGCG was shown to inhibit HSV infection by blocking binding of the virions to cellular receptors, we tested the effect of EGCG-AgNPs against HSV-1/HSV-2 attachment to the host cell surface and subsequent membrane fusion in two types of human keratinocytes—skin-derived HaCaT cells and vaginal VK-2-E7/E7 cells. The details of the procedure are described in Materials and Methods. We observed that EGCG-AgNPs were significantly better at inhibiting HSV-1 and HSV-2 attachment and penetration for both cell lines compared to EGCG itself (*p* ≤ 0.01) (Figure 3). However, the latter also blocked viral attachment and penetration in a significant manner (approx. 40%, *p* ≤ 0.05) (Figure 3). Furthermore, EGCG-AgNPs were significantly more efficient in blocking HSV-1 and HSV-2 penetration into the HaCaT keratinocytes and HSV-1 and HSV-2 attachment to VK-2 cells compared to TA-AgNPs (*p* ≤ 0.01) (Figure 3). 

### 3.3. EGCG-Modified AgNPs Reduce Murine HSV-1 and HSV-2 Infection In Vivo by Stimulation of Immune Response

**Figure 4:**
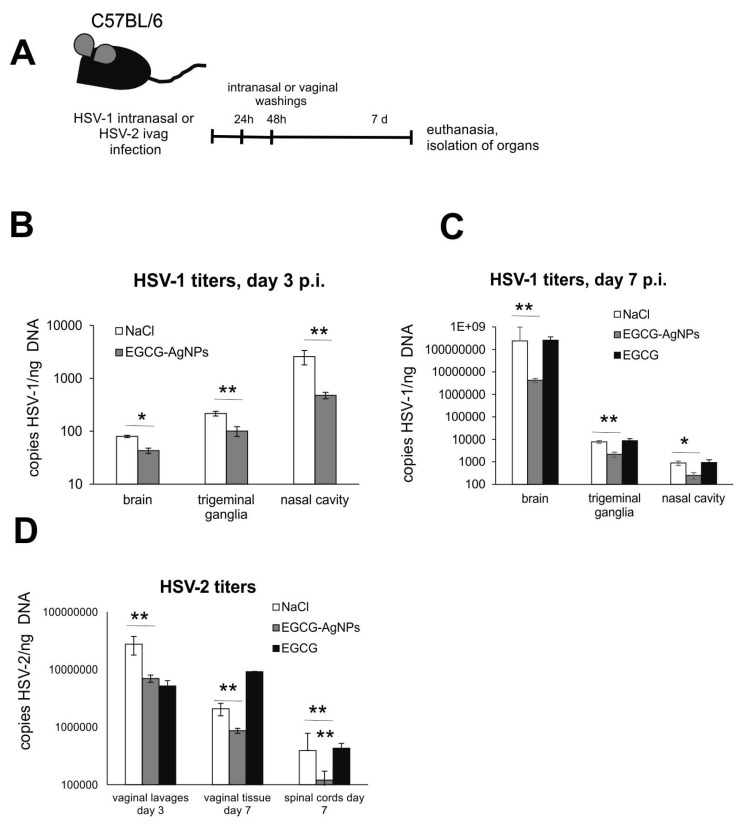
Treatment with epigallocatechin gallate-modified AgNPs reduce HSV-1 and -2 infection in vivo. (**A**) C57BL/6 mice infected intranasally with HSV-1 or intravaginally with HSV-2 were treated twice every 24 h with EGCG or EGCG-AgNPs. Trigeminal ganglia, nasal cavities and brains collected at 3 (**B**) and 7 days p.i. (**C**), as well as vaginal lavages, vaginal tissues and spinal cords isolated at 7 d p.i. (**D**), were subjected to measurement of HSV-1 or -2 gB titers (copies/ng DNA) by qPCR (N = 10). The bars represent means ± SEM. * represents significant differences with *p* ≤ 0.05, ** *p* ≤ 0.01 in comparison to untreated infected tissues.

To confirm our in vitro findings, we employed two well-established murine models of HSV-1 and HSV-2 infection [17,18]. In general, EGCG-AgNPs were used as antiviral agent in animals with on-going intranasal (HSV-1) or intravaginal (HSV-2) primary infection (Figure 4). EGCG was used as a control, in a concentration corresponding to the amount used to modify AgNPs (200 μM).

#### 3.3.1. HSV-1 Infection Model

To study how local application of EGCG-AgNPs can be used for treatment of HSV-1 infection, we applied intranasally 20 μL of 20 μg/mL of 30 nm EGCG-AgNPs at 24 and 48 h p.i. (Figure 4). At 3 and 7 days p.i., we measured HSV-1 titers in trigeminal ganglia (TGs), nasal cavities and brains of mice treated with EGCG, EGCG-AgNPs or NaCl (Figure 4B,C, respectively). We found that intranasal treatment with EGCG-AgNPs at the early stage of infection led to significantly lower titers of HSV-1 in all tested organs at 3 and 7 days p.i. (*p* ≤ 0.05) (Figure 4B,C).

#### 3.3.2. HSV-2 Infection Model

Next, we used the intravaginal HSV-2 model to study the effects of post-infection treatment of the vaginal epithelium. The mice were infected with HSV-2 and then the vaginas were irrigated twice with 100 μL of 20 μg/mL of 30 nm EGCG-AgNPs, EGCG or NaCl at 24 and 48 h p.i. Next, we measured HSV-2 titers with qPCR in the vaginal tissues and spinal cords isolated at 7 days p.i. and in the vaginal lavages collected at 48 h p.i. (Figure 4D). Post-infection treatment with EGCG-AgNPs resulted in a significant decrease of the viral titers in vaginal lavages, vaginal tissues, and spinal cords (*p* ≤ 0.01) (Figure 4D).

#### 3.3.3. EGCG-Modified AgNPs Help to Activate Early Antiviral Response in HSV-1 Infection

**Figure 5:**
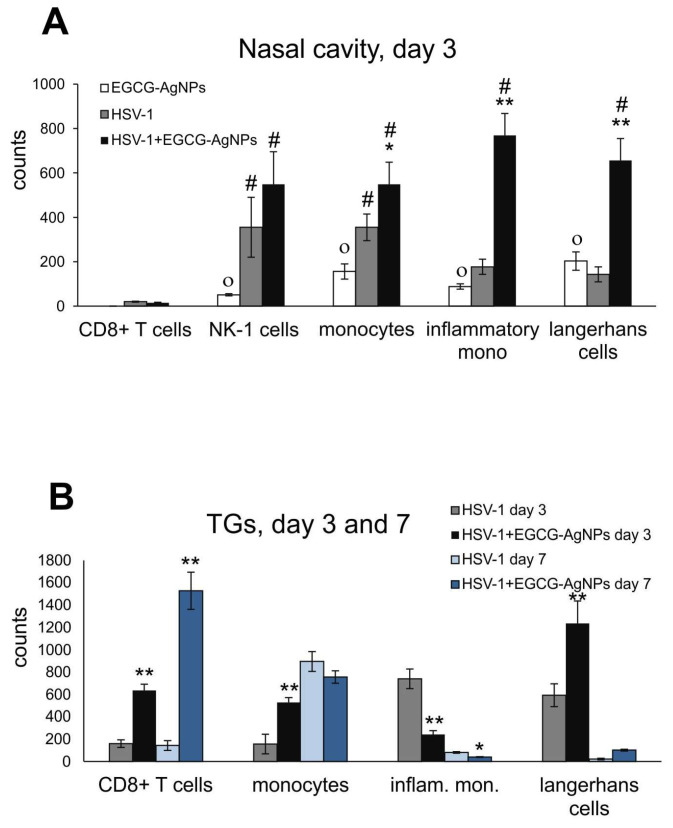
EGCG-modified AgNPs activate early antiviral response in HSV-1 nasal infection. Total counts of CD8+ T cells, NK cells, monocytes, inflammatory monocytes, and Langerhans cells in the uninfected and HSV-1-infected nasal cavity treated with EGCG-AgNPs and isolated at 3 days p.i. (**A**), and in the trigeminal ganglia (TGs) isolated at 3 and 7 days p.i. (**B**). Results are expressed as mean ± SEM for N = 7. * Represents significant differences with *p* ≤ 0.05, ** *p* ≤ 0.01 in comparison to HSV-1 infected tissues, ° represents significant differences with *p* ≤ 0.05 in comparison to uninfected tissues, # represents significant differences with *p* ≤ 0.05 in comparison to uninfected tissues treated with EGCG-AgNPs.

Together with viral titers in HSV-1/2 infected animals, we tested if EGCG-modified AgNPs can contribute to activation of early antiviral immune response during HSV-1 infection, as previously demonstrated for TA-AgNPs [17,18]. Cell suspensions prepared from nasal cavities, trigeminal ganglia (TGs) and whole brains were subjected to immunophenotyping for CD4+ T cells, CD8+ T cells, NK cells, Langerhans cells, monocytes and inflammatory monocytes (Figure 5). Treatment with EGCG-modified AgNPs of uninfected nasal cavities resulted in significantly up-regulated infiltration of NK-1 cells, monocytes, inflammatory monocytes, and Langerhans cells compared to uninfected nasal cavities (*p* ≤ 0.05) (Figure 5A). Infection with HSV-1 also led to significant up-regulation of CD8+ T cells, NK-1 cells, monocytes, inflammatory monocytes, and Langerhans cells at 3 days p.i. compared to uninfected control (*p* ≤ 0.05) (Figure 5A). During infection with HSV-1, only monocytes and NK-1 cells were significantly up-regulated compared to EGCG-AgNP treated uninfected mucosal tissues (*p* ≤ 0.05) (Figure 5A). Interestingly, post-infection treatment with EGCG-AgNPs significantly increased counts of monocytes, inflammatory monocytes, and Langerhans cells at 3 days p.i. in comparison to infected untreated nasal cavities (*p* ≤ 0.05) (Figure 5A). 

Identification of infiltrating immune competent cells in TGs at 3 and 7 days p.i. demonstrated that treatment with EGCG-AgNPs significantly increased numbers of CD8+ T cells at both time points compared to untreated infected tissues (*p* ≤ 0.01) (Figure 5B). Importantly, EGCG-AgNPs treatment induced significant infiltration of monocytes and Langerhans cells early during infection (3 days p.i.) (*p* ≤ 0.01), but not of inflammatory monocytes (Figure 5B), the infiltration of which was significantly decreased (*p* ≤ 0.05) (Figure 5B) compared to untreated tissues. In contrast to nasal cavities and TGs, brains at 7 days p.i. showed significantly decreased infiltration of CD8+ T cells, CD8+/IFN-gamma+ T cells and NK cells in brains isolated from EGCG-AgNP treated mice (Supplementary Figure S1). No significant increase of any cell type was detected in TGs and brains of uninfected, control mice treated with EGCG.

To understand how EGCG-AgNPs influence the local concentration of antiviral cytokines and chemokines, we measured expression of IFN-4α, IFN-β, CXCL1, CXCL9, CXCL10, IL-1β, IL-10, IL-6 and TNF-α mRNAs in primary murine keratinocytes infected with HSV-1 (Supplementary Figure S2A). Keratinocytes were first infected for 4 h, then treated with 7.5 μg/mL of EGCG-AgNPs for the next 20 h. At 24 h p.i., we did not detect TNF-α and no significant up-regulation of IL-10 was found (Supplementary Figure S2A). However, uninfected control cells treated with EGCG-AgNPs showed significant upregulation of CXCL9, CXCL10, IL-1β, IL-6, IFN-4α and IFN-β mRNA expression in comparison to untreated control cells (*p* ≤ 0.05). The same treatment of HSV-1 infected keratinocytes also increased CXCL1, CXCL9, CXCL10, IFN-β mRNA expression levels (*p* ≤ 0.05), while it decreased mRNA expression for IL-6 (*p* = 0.045) compared to infected untreated control (Supplementary Figure S2A). 

**Figure 6:**
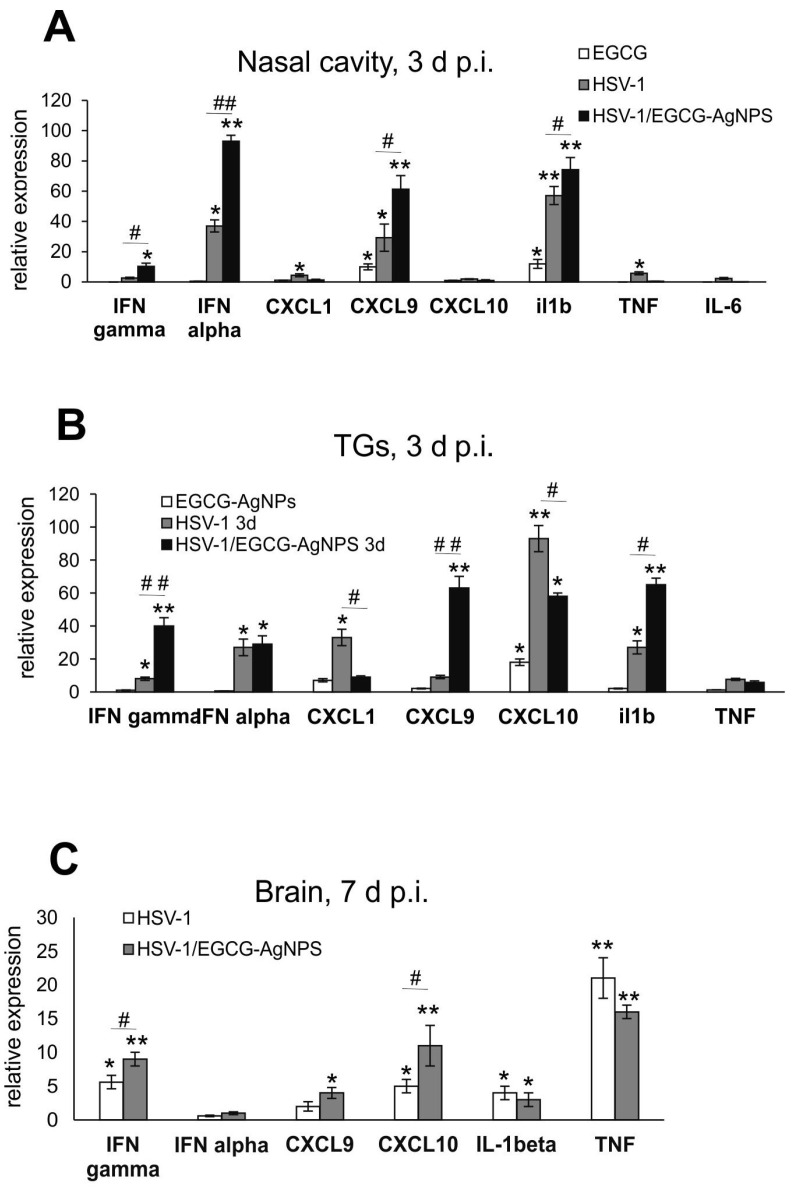
Cytokine and chemokine expression in the nasal cavities, trigeminal ganglia and brains at 3 or 7 days p.i. after treatment with EGCG-AgNPs. Levels of IFN-α, IFN-γ, CXCL1, CXCL9, CXCL10, IL-1β, TNF-α mRNAs are shown as expression relative to control based on the 2^−∆∆Ct^ method. N = 7. * Represents significant differences with *p* ≤ 0.05, ** *p* ≤ 0.01 in comparison to uninfected tissues, # represents significant differences with *p* ≤ 0.05, ## *p* ≤ 0.01 in comparison to infected but untreated tissues.

Upon analysis of the in vivo HSV-1 infection model, we observed a similar expression trend. In the nasal cavity, only IL-1β and CXCL9 mRNA expression levels were significantly upregulated in uninfected, EGCG-AgNPs-treated mucosal tissues (*p* ≤ 0.05) (Figure 6A). Treatment of HSV-1 nasal infection resulted in a significant increase of IFN-4α, IFN-γ, CXCL9 and IL-1β mRNA expression (*p* ≤ 0.05) (Figure 6A). In TGs, the use of EGCG-AgNPs in uninfected controls resulted in a significant up-regulation of CXCL10 mRNA expression (*p* = 0.039) (Figure 6B), while upon infection at 3 days p.i., EGCG-AgNPs up-regulated mRNA expression levels for IFN-γ, CXCL9 and IL-1β (*p* ≤ 0.05), and down-regulated mRNA expression levels for CXCL10 and CXCL1 (*p* ≤ 0.05) (Figure 6B). The brains of HSV-1 infected and EGCG-AgNPs-treated mice showed significant up-regulation of CXCL10 and IFN-γ in comparison to untreated infected mice at 7 days p.i. (*p* ≤ 0.05) (Figure 6C). No up-regulation of any cytokine or chemokine was observed for control, EGCG-AgNP-treated animals.

#### 3.3.4. EGCG-Modified AgNPs Help to Activate Early Antiviral Response in HSV-2 Vaginal Infection

Since HSV-2 infection also starts at the mucosa, we employed a murine HSV-2 model of genital infection to further study the effects of EGCG-AgNP treatment upon activation of early mucosal antiviral response. Cell suspensions prepared from the vaginal tissues at 3 and 7 days p.i. were first tested for infiltration of dendritic cells and monocytes.

**Figure 7:**
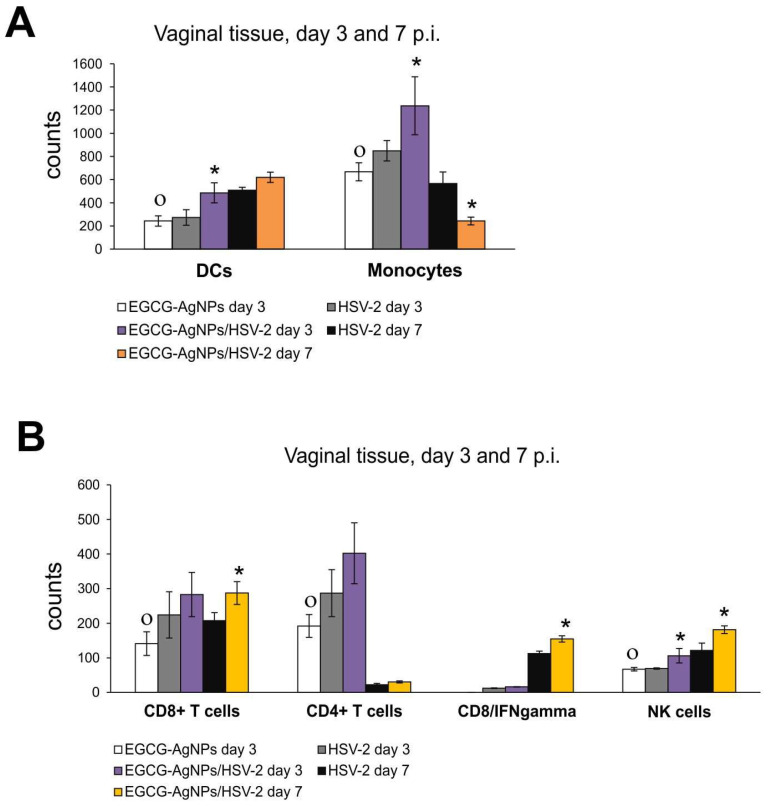
EGCG-modified AgNPs activate early antiviral response in HSV-2 vaginal infection at 3 and 7 days p.i. Total counts of (**A**) monocytes and dendritic cells, (**B**) CD4+ T cells, CD8+ T cells, CD8+/IFN-γ, NK cells in the uninfected and HSV-2-infected vaginal tissue treated with EGCG-AgNPs. Results are expressed as mean ± SEM for N = 7. * Represents significant differences with *p* ≤ 0.05 in comparison to HSV-1 infected tissues, ° represents significant differences with *p* ≤ 0.05 in comparison to uninfected tissues.

Both cell types were up-regulated in EGCG-AgNP-treated uninfected controls compared to untreated controls (*p* ≤ 0.05) (Figure 7A), while in HSV-2 infected mucosal tissue, EGCG-AgNPs induced a significant infiltration of DC monocytes only at 3 days p.i. (*p* ≤ 0.05) (Figure 7A). On the other hand, the same treatment significantly reduced monocyte infiltration later during infection (7 days p.i.) (*p* ≤ 0.05) (Figure 7A). Identification of other infiltrating immune competent cells in vaginal tissues demonstrated that treatment with EGCG-AgNPs of uninfected vaginal mucosa significantly increased numbers of CD4+ T cells, CD8+ T cells and NK cells at 3 days p.i. compared to untreated control tissues (*p* ≤ 0.05) (Figure 7B). Upon EGCG-AgNPs treatment, HSV-2 infected vaginal tissues were significantly infiltrated with CD4+ T cells, CD8+ T cells, CD8+ T/IFN-γ T cells both at 3 and 7 days p.i. in comparison to untreated infected tissues, but these differences were significant only at 7 days p.i. (*p* ≤ 0.05) (Figure 7B).

To further elucidate the local antiviral response upon EGCG-AgNP treatment, we employed similar in vitro model of primary keratinocytes infected with HSV-2 and treated with EGCG-AgNPs 4 h later (Supplementary Figure S3). We did not detect TNF-α (not shown) and no significant up-regulation of IL-10 was found (Supplementary Figure S2). Treatment of HSV-2 infected keratinocytes increased CXCL1, CXCL9, CXCL10, IFN-β mRNAs expression levels (*p* ≤ 0.05), while there was decreased mRNAs expression for IL-1β, IL-6 (*p* ≤ 0.05) (Supplementary Figure S3).

**Figure 8:**
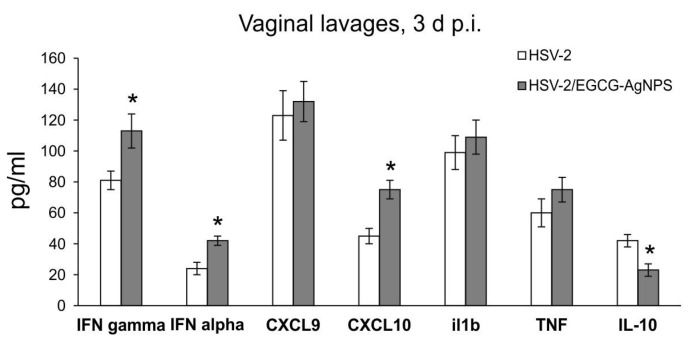
Cytokine and chemokine production in HSV-2 infected vaginal tissues at 3 days p.i., treated or untreated with EGCG-AgNPs. Levels of IFN-α, IFN-γ, CXCL9, CXCL10, IL-1β, TNF-α and IL-10 are shown in pg/mL. N = 7. * Represents significant differences with *p* ≤ 0.05, in comparison to infected but untreated tissues.

Analysis of a HSV-2 genital infection model using lavages obtained at 3 days p.i. showed no detectable levels of the tested cytokines or chemokines in EGCG-AgNP-treated uninfected controls. Figure 8 demonstrates significant results obtained for vaginal lavages from HSV-2 infected mice, treated or untreated with EGCG-AgNPs. EGCG-AgNPs significantly induced levels of IFN-α and IFN-γ, as well as of CXCL10 in the vaginal lavages, compared to untreated HSV-2 infected mice (*p* ≤ 0.05) (Figure 8). Additionally, EGCG-AgNP treatment significantly decreased production of IL-10 in the vaginal tissues (*p* ≤ 0.05) (Figure 8).

## 4. Discussion

Recent COVID-19 pandemic, as well as the constant threat of newly emerging and re-emerging viruses, have shown an urgent need for broad-acting, non-toxic antivirals that reduce the virus load in the body or on surfaces. Our rationales for studying the anti-HSV activity of EGCG-modified AgNPs were based on the following: (1) EGCG has a broad-spectrum antiviral effect on both RNA and DNA viruses [6,7,8,9,10,11,12]; (2) AgNPs with functionalized surface also show strong anti-viral activity [15,16,17,18,19]; (3) EGCG can significantly reduce inflammation processes [1,2,3,4,5].

EGCG forms water-soluble complexes with many proteins, indicating the existence of certain binding patterns [6,7]. The studies of antiviral activity against different viruses showed that EGCG acts directly on the virion surface proteins and blocks the primary low-affinity attachment of virions to cells, without affecting the fluidity or integrity of the virion structure [7].

The HSV virion contains multiply glycoproteins. Two of these glycoproteins—gB and gC—bind heparan sulfate on a cell surface, followed by viral gD binding to entry receptors such as herpes virus entry mediator (HVEM). This leads to conformational changes, formation of a complex with other viral glycoproteins, H (gH) and L (gL), and further virus entry into the cell. Studies by Isaacs et al. (2008) demonstrated that EGCG inactivates multiple clinical isolates of HSV-1 and HSV-2 [6]. Tests with purified gB and gD showed that EGCG can form macromolecular complexes with viral glycoproteins, preventing virion fusion with cell membranes [6]. Furthermore, electron microscopic (EM) imaging showed that exposition of virions to EGCG caused damage, with EM immunogold labeling indicating binding of EGCG with gB and gD [6]. EGCG can inactivate multiple strains of HSV-1 (strain F1, 17 syn+, KOS) and HSV-2 (strain 333), which indicates a universal mechanism of antiviral action for HSV viruses [6,7,21].

Here, we found that both EGCG and EGCG-AgNPs are efficiently inhibiting HSV-1 strain McKrae and HSV-2 strain 333, although EGCG-AgNPs were at least twice as efficient as antivirals in comparison to EGCG solution at the same concentration (Figure 2 and Figure 3). We have also previously shown that tannic acid-modified AgNPs are much more efficient as antivirals compared to tannic acid itself [17,18,19]. Tannic acid (TA) is a plant polyphenol, which can be treated as a gallic acid polymer due to multiply gallic acid moieties in its structure [22]. TA is obtained from plants, but its structure and the amount of gallic acid residues is difficult to predict. On the other hand, EGCG can be easily either isolated or synthesized to obtain a pharmaceutical ingredient. Tannic acid-modified AgNPs sized 30 nm inhibited HSV-1 and HSV-2 infection in HaCat human keratinocyte cell line [23]. TA-AgNPs interacted directly with the virion surface, influencing viral attachment, binding and cell-to-cell spread [17,18]. Here, we found that EGCG is effective in blocking HSV penetration and attachment in HaCaT and VK-2 keratinocyte cell lines, but functionalization of AgNPs with EGCG increases its anti-HSV activity (Figure 3), also in comparison to TA-AgNPs.

Interestingly, EGCG-AgNPs were more efficient than TA-AgNPs in inhibition of viral penetration in HaCaT cells and inhibition of viral attachment in VK-2 cells (Figure 3). This tendency was observed for both HSV-1 and HSV-2, which indicates that this difference is probably cell-type related and depends on heparan sulphate structure and other cellular receptors present on these two cell types. Nevertheless, EGCG-AgNPs are much more effective as antivirals, probably due to the mechanism of binding with the virion’s surface.

Reshamwala et al. (2021) used both resveratrol and EGCG to functionalize gold nanoparticles (AuNPs) and tested their antiviral efficiency, showing reduction of infection with three enterovirus serotypes and a better toxicology profile for EGCG-AgNPs compared to EGCG [24]. Both RES- and EGCG-AuNPs exerted a direct effect through binding to the virus particles, preventing the virus from binding to the cell surface and inducing clustering of the virions [24]. Computational modelling showed that EGCG immerses in several binding sites on the virion surface, resulting in stabilization and aggregation of the virions [24]. Therefore, we can conclude that functionalization of Ag/AuNPs with EGCG opens a possibility of developing a new antiviral, which has a universal action mode against several viruses by interfering with virus binding to the target cells.

There are several reports showing the efficacy of EGCG as an antiviral drug in vivo. Mice intranasally infected with OC43 (HCoV-OC43) as a surrogate for SARS-CoV-2 received 10 mg/kg body weight EGCG daily for 2 weeks via regular drinking bottle. Virus titers in the lung tissue were significantly reduced in animals receiving EGCG or green tea extract [25]. Also, 40 mg/kg EGCG provided complete protection in BALB/c mice infected with pseudorabies virus (PRV) XJ5, when EGCG was administrated both pre- and post-infection [26].

Considering the results obtained in vitro, we decided to employ EGCG-AgNPs to treat in vivo models of HSV-1 and HSV-2 infection. We found that the amount of EGCG used to modify AgNPs was not sufficient to protect mice from either HSV-1 or HSV-2 infection, while the same amount bound to nanoparticles resulted in a good antiviral activity (Figure 4). Some authors postulate that high amounts of EGCG exert in vitro hepatotoxic effects, and dermo-toxicity in vivo [27,28]. Therefore, we can assume that modification of AgNPs with much lower amounts of EGCG provides a better toxicological profile and selectivity index, as discussed in [24].

As mentioned above, EGCG can also reverse the virus-induced down-regulation of the type I interferon (IFN) signaling pathway leading to the transcription of interferon-stimulated genes (ISGs), necessary for an efficient anti-viral response [12]. Furthermore, we previously demonstrated that both TA-AgNPs and lactoferrin-modified AgNPs can stimulate migration of dendritic cells (DCs) into the vaginal tissue, not only during primary infection [18,19], but also later, upon re-challenge with the virus [18]. Modified NPs helped to present the virus antigens both to naïve cytotoxic T cells in the lymph nodes and to memory T cells resulting in the activation of the specific antiviral response [18]. In this study, we also observed significant migration of monocytes and Langerhans cells to both infected and uninfected mucosal tissues of the nasal cavity (Figure 5), which was further reflected by increased infiltration of CD8+ T cells into infected TGs (Figure 5). Furthermore, EGCG-AgNPs significantly increased expression of cytokines and chemokines important in anti-HSV response (IFN-α, IFN-γ, CXCL9, CXCL10) (Figure 6 and Figure 8).

Similar effects of EGCG-AgNP treatment were observed in HSV-2 infected vaginal tissues, where NPs stimulated migration of both monocytes and dendritic cells (Figure 7). Interestingly, EGCG-AgNPs reduced monocyte infiltration later during infection, most probably due to efficient virus eradication and mounting of an antiviral response by CD8+ T cells and NK cells (Figure 7). We may conclude that EGCG-AgNPs can provide a good adjuvant to boost the anti-viral immune response. If we assume that EGCG helps to bind virus surface, leading to accumulation of aggregates of modified NPs with HSV at the site of infection, as suggested by our and Remshawala’s studies [18,19,24], viral antigens may be better internalized and processed by antigen-presenting cells (APCs). This in turn leads to better activation of immune competent cells downstream. The mechanistical role of EGCG in binding viral antigens needs further elucidation, but silver and gold nanoparticles as adjuvants for infectious agents are attracting attention [29].

Furthermore, the study by Cheong et al. presented EGCG as a novel vaccine adjuvant for viral vaccines with an effect synergistic with alum [30]. The coadministration of epigallocatechin-3-gallate (EGCG) with influenza hemagglutinin (HA) antigens induced high levels of neutralizing antibodies in mice, comparable to those induced by alum. In this manner, mice were provided with a complete protection against the lethal challenge with a live virus [30]. Additionally, EGCG induced immunoglobulin isotype switching from IgG1 to IgG2a, leading to a more balanced Th1/Th2 response compared to alum [30]. One cannot exclude that EGCG also stimulated cellular pathways related to interferon-induced genes and antiviral response.

In this study, the adjuvant activity of EGCG may be synergistic to AgNPs. Silver nanoparticles sized 30 nm have good surface to volume ratio and provide a vast surface for virus–EGCG interaction. This in turn leads to creation of aggregates, recognized by APCs. Further studies are necessary to elucidate the exact mechanism of EGCG-AgNPs’ interaction with virus surface and their possible role as adjuvants.

## 5. Conclusions

In the present study, we demonstrated that EGCG-modified AgNPs sized 30 nm inhibit HSV-1 and HSV-2 infection both in vitro and in vivo. The inhibitory effect of EGCG-AgNPs on the HSV-1/2 was much more effective than EGCG solution at the same concentration. We subsequently demonstrated that EGCG-modified AgNPs were acting on HSV by blocking its replicative cycle at the attachment and penetration stage. Apart from their direct antiviral activity, EGCG-AgNPs stimulated efficient antiviral response in murine models of mucosal infection with HSV-1 or HSV-2. Therefore, the dual function of EGCG, as a virucidal and an adjuvant agent, could be used to develop an effective microbicide applied on the mucosal tissues.

## Figures and Tables

**Table 1:** Results of AgNPs, EGCG-AgNPs and TA-AgNPs characterization by localized surface plasmon resonance (LSPR), HR-STEM and Zeta potential measurements.

	d_STEM_ [nm]	d_H_ [nm]	λmax [nm]	Zeta Potential [mV]
AgNPs	26 ± 4	30 ± 10	395	−76 ± 1
EGCG-AgNPs	26 ± 4	32 ± 11	400	−67 ± 2
TA-AgNPs	27 ± 7	35 ± 10	407	−58 ± 2

**Table 2:** Cytotoxicity of EGCG-modified and unmodified 30 nm AgNPs or corresponding EGCG solution in Vero, VK-2, primary keratinocytes and HaCaT cells ^1^.

	EGCG ^4^	30 nm EGCG-AgNPs	30 nm AgNPs
Vero cells CC_50_ ^2^ (µg/mL ± SEM)	45.02 ± 6.9	39.02 ± 7.3	30.8 ± 3.4
Vero cells MNTC ^3^ (µg/mL ± SEM)	16.99 ± 2.3	15.22 ± 5.2	10.5 ± 2.4
HaCaT cells CC_50_ ^2^ (µg/mL ± SEM)	38.8 ± 5.1	35.3 ± 1.9	33.3 ± 5.1
HaCaT cells MNTC ^3^ (µg/mL ± SEM)	15.77 ± 2.5	12.33 ± 1.4	9.02 ± 0.5
VK-2 cells CC_50_ ^2^ (µg/mL ± SEM)	37.55 ± 6.8	33.8 ± 0.8	27.54 ± 1.8
VK-2 cells MNTC ^3^ (µg/mL ± SEM)	14.9 ± 3.1	12.44 ± 4.3	9.8 ± 2.2
Primary keratinocytes CC_50_ ^2^ (µg/mL ± SEM	32.1 ± 1	29.99 ± 4.9	24.66 ± 5.2
Primary keratinocytes MNTC ^3^ (µg/mL ± SEM)	11.3 ± 1.99	11.5 ± 3.0	9.87 ± 2.1

^1^ The values shown are means from three independent experiments with each treatment performed in triplicate ± SEM. ^2^ Cytotoxic effects were evaluated by MTT assay to determine the concentration of 50% cellular cytotoxicity (CC_50_) of the tested compound and calculated by regression analysis, plotting cytotoxicity percentage to NP concentration. ^3^ MNTC (maximum non-toxic concentrations) was calculated as NP concentration with ≤20% of non-viable cells. ^4^ EGCG solution in concentration corresponding to concentration present in colloids (200 μM).

## Data Availability

Data available from authors at request.

## Supplementary Materials

The following supporting information can be downloaded at: https://www.mdpi.com/article/10.3390/v15102024/s1, Figure S1: Antiviral response in brains of HSV-1 infected mice treated or untreated with EGCG-modified AgNPs; Figure S2: Cytokine and chemokine expression changes in the primary murine keratinocytes infected with HSV-1; Figure S3: Cytokine and chemokine expression changes in the primary murine keratinocytes infected with HSV-2.
