# Supplementary material for: Epigallocatechin Gallate-Modified Silver Nanoparticles Show Antiviral Activity against Herpes Simplex Type 1 and 2
Source: Viruses. 2023 Sep 29;15(10):2024. doi: 10.3390/v15102024 (PMC10611064; doi:10.3390/v15102024)
Supplement: Supplementary file 1 [file viruses-15-02024-s001.zip › viruses-2619331-supplementary.pdf]

Supplementary Figures

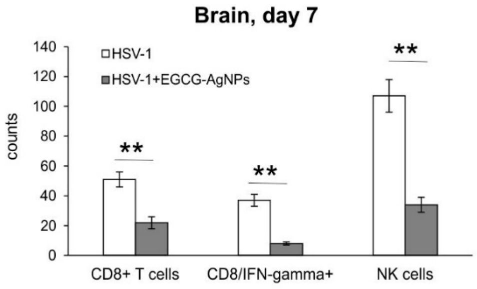

Supplementary Figure S1. Antiviral response in brains of HSV-1 infected mice treated or untreated with EGCG-modified AgNPs. Total counts of CD8+ T cells, CD8+/IFN-gamma+ T cells and NK cells, in brains isolated at 7 day p.i., Results are expressed as mean +/− SEM for N = 7. ** represents significant differences with $p \leq 0.01$.

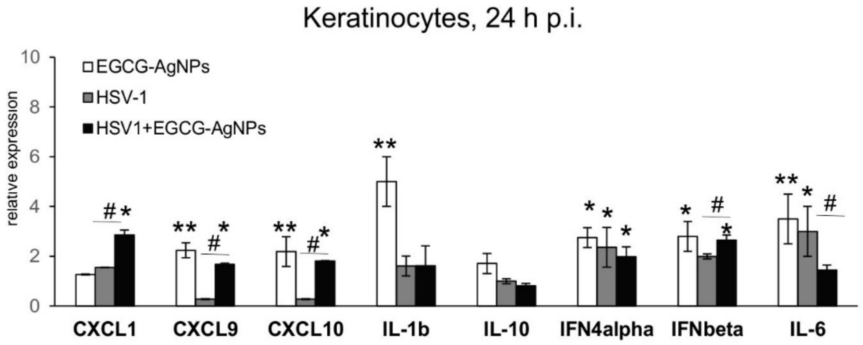

Supplementary Figure S2. Cytokine and chemokine expression changes in the primary murine keratinocytes infected with HSV-1. Levels of CXCL1, CXCL9, CXCL10, IL-1β, IL-10, IFN-4α, IFN-β and IL-6 mRNAs are shown as expression relative to control based on the $2^{-\Delta\Delta Ct}$ method. N = 3. * represents significant differences with $p \leq 0.05$, ** $p \leq 0.01$ in comparison to untreated HSV-2 tissues, # represents significant differences with $p \leq 0.05$, compared to infected but untreated tissues.

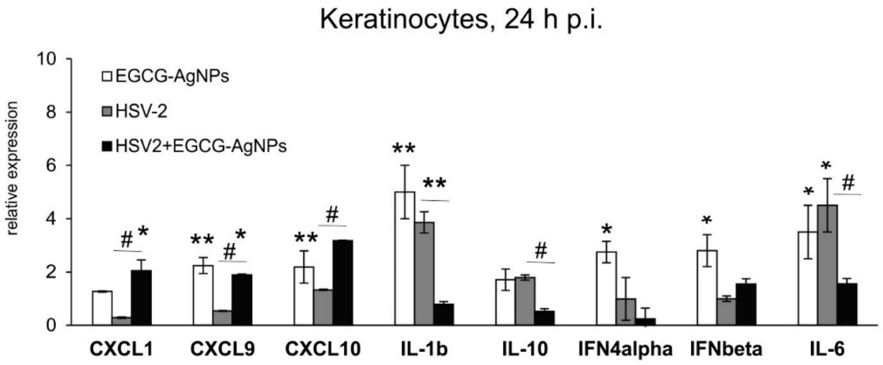

Supplementary Figure S3. Cytokine and chemokine expression changes in the primary murine keratinocytes infected with HSV-2. Levels of CXCL1, CXCL9, CXCL10, IL-1β, IL-10, IFN-4α, IFN-β and IL-6 mRNAs are shown as expression relative to control based on the $2^{-\Delta\Delta Ct}$ method. N = 3. * represents significant differences with p ≤

0.05, ** $p \leq 0.01$ in comparison to untreated HSV-2 tissues, # represents significant differences with $p \leq 0.05$, compared to infected but untreated tissues.
